# Intelligent Localization of Cross-Sectional Structural Damage in Molten Salt Receiver Tubes Using Mel Spectrograms and TSA-Optimized 2D-CNN

**DOI:** 10.3390/s26092780

**Published:** 2026-04-29

**Authors:** Peiran Leng, Man Liang, Weihong Sun, Tiefeng Shao, Luowei Cao, Sunting Yan

**Affiliations:** 1 School of Mechanical and Electrical Engineering, China Jiliang University, Hangzhou 310018, China; lpr523200794@163.com (P.L.); whsun@cjlu.edu.cn (W.S.); stf@cjlu.edu.cn (T.S.); 2 China Special Equipment Inspection and Research Institute, Beijing 100029, China; caoluowei@csei.org.cn; 3 Zhejiang Academy of Special Equipment Science, Hangzhou 310018, China; yansunting@zju.edu.cn; 4 Zhejiang Key Laboratory of Special Equipment Safety Technology, Hangzhou 310018, China

**Keywords:** molten salt receiver tube, ultrasonic guided wave defect localization, two-dimensional convolutional neural network (2D-CNN), Mel spectrogram, Tree Seed Algorithm (TSA)

## Abstract

In this paper, an intelligent localization framework based on deep learning is proposed to address the limitations of insufficient accuracy and robustness in defect identification and localization during the ultrasonic guided-wave non-destructive testing (NDT) of receiver tubes in tower-type molten salt Concentrated Solar Power (CSP) stations. In the proposed method, a 1D convolutional neural network (1D-CNN) initially processes raw time-series-guided wave signals, achieving coarse identification and preliminary localization of defective segments. Then, Mel spectrograms are employed to exploit multi-dimensional features in the time–frequency domain and transform 1D signals into 2D representations, thereby enriching feature diversity. A regression-based 2D-CNN was designed to predict the start and end points of defect segments, enabling precise interval localization. Furthermore, the Tree Seed Algorithm (TSA) was integrated to jointly optimize key hyperparameters, enhancing training efficiency and prediction accuracy. Experimental validation on a dataset of ultrasonic guided-wave signals from molten salt receiver tubes demonstrates that the TSA-optimized Mel+2D-CNN model achieves superior performance, with a Mean Absolute Error (MAE) of 75.11 sampling points and a Coefficient of Determination (R^2^) of 0.90. At an Intersection over Union (IoU) threshold of 0.3, the model achieves a hit rate of 89.21%, exhibiting significantly higher localization accuracy and stability compared to the 1D-CNN baseline model. These findings indicate that the proposed method effectively enhances the accuracy and robustness of guided wave-based defect localization in slender structures. While promising, the model’s generalization capability remains dependent on the data distribution and operating conditions; future work will focus on validating its engineering applicability across diverse, multi-scenario industrial environments.

## 1. Introduction

Concentrated Solar Power (CSP) plants, particularly tower-type systems that utilize molten salt as a heat transfer fluid, are critical for large-scale renewable energy generation due to their high-efficiency thermal energy storage and conversion. The receiver tube, a key component in these systems, is subjected to extreme operational conditions—including prolonged exposure to high temperatures, high pressures, corrosive molten salts, and cyclic thermal–mechanical loads—making it susceptible to cross-sectional damage such as fatigue cracks, thermal stress fractures, and corrosion. Traditional inspection methods, which rely on manual examination, are not only labor-intensive and costly but also inadequate for real-time structural health monitoring. Given that receiver tube failure could lead to severe safety hazards and substantial economic losses, there is a pressing need for efficient and accurate non-destructive evaluation techniques [1,2,3].

Ultrasonic guided wave testing has emerged as a promising approach for inspecting pipelines and slender structures, owing to its long-range propagation capability and adaptability to coated surfaces. In practical field environments, however, guided wave signals are often contaminated by coherent noise from factors such as non-uniform salt deposition and surface roughness, which complicates the reliable detection and localization of defect echoes. To address these challenges, deep learning methods have been increasingly applied to guided wave signal analysis. One-dimensional convolutional neural networks (1D-CNNs), for instance, have been widely adopted for defect classification and sizing. Early work by Wang et al. [4] demonstrated the feasibility of integrating deep learning with ultrasonic guided wave data. Subsequent studies by Kim et al. [5] and Ma et al. [6] further advanced the accuracy of defect characterization under complex environmental conditions. For scenarios with limited labeled data, researchers such as Wang et al. [7] and Tang et al. [8] have employed fully convolutional networks and transfer learning strategies to maintain robust performance with small datasets. Parallel efforts have focused on model efficiency and interpretability, with contributions from Sun et al. [9] in model lightweighting and from Wang et al. [10] and Jiang et al. [11] in physics-guided neural network architectures. To better capture the non-stationary and dispersive nature of guided waves, several studies have explored transforming one-dimensional time-series signals into two-dimensional time–frequency representations. Techniques involving, for example, Gramian Angular Fields (GAFs) [12], Mel spectrograms [13], and the Pseudo Wigner–Ville Distribution (PWVD) [14] have been used to generate rich feature maps that enhance defect-related pattern recognition. Furthermore, the optimization of hyperparameters—a critical step in deep learning model development—has benefited from intelligent search algorithms. Methods such as the Tree Seed Algorithm (TSA) [15], the Golden Sine Algorithm [16], and enhanced swarm intelligence approaches [17] have been successfully applied to automate hyperparameter tuning, reducing reliance on manual search and improving model generalization.

Despite these advances, several research gaps remain:

(1) Regression capability: While 1D-CNNs excel in classification tasks [18], they are less effective for regression-based defect localization, primarily due to their limited ability to jointly model time and frequency domain features.
(2) Time–frequency representation: Although two-dimensional transforms improve feature richness, the potential of perceptually motivated representations such as the Mel spectrogram [19]—particularly for capturing guided wave dispersion under noisy conditions—has not been fully explored in structural health monitoring.
(3) Hyperparameter optimization: Conventional methods like grid search are computationally expensive and prone to suboptimal solutions, especially in high-dimensional parameter spaces required for regression models.

**Figure 1:**
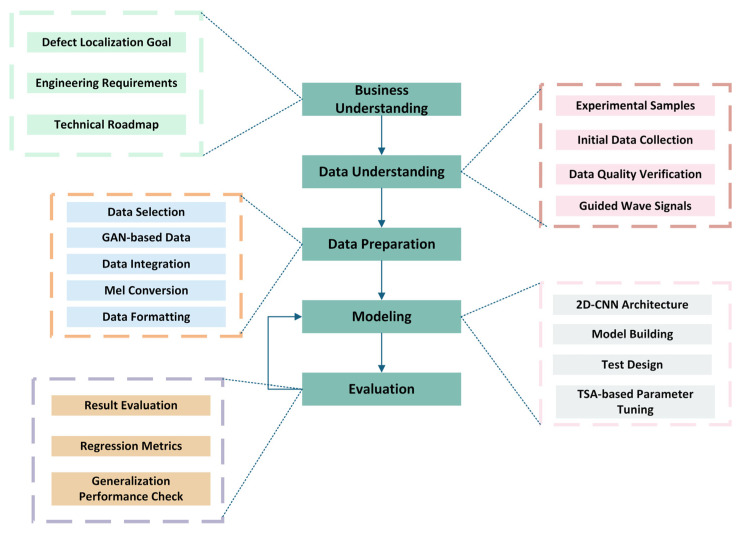
Workflow of the TSA-Mel-2DCNN-based defect localization model for receiver tubes.

To bridge these gaps, this paper presents an intelligent localization framework for cross-sectional damage in molten salt receiver tubes. The proposed method employs Mel spectrograms to convert guided wave signals into time–frequency images, processes them using a 2D-CNN, and optimizes the network hyperparameters using the Tree Seed Algorithm. By integrating a perceptually relevant time–frequency representation with an adaptive optimization strategy, the model achieves accurate regression of defect intervals, offering a robust and automated solution for structural health monitoring in CSP systems. Thus, we have developed an overall experimental workflow by adapting the CRISP-DM framework [20] to the specific requirements of our NDT task, as shown in Figure 1.

The main contributions of this work are summarized as follows:

- A Mel spectrogram-based 2D representation method for guided wave signals. To overcome the limitations of 1D-CNNs in capturing time–frequency characteristics from raw time-series signals, we convert one-dimensional guided wave signals into Mel spectrogram images. This transformation provides a richer time–frequency representation that enhances subsequent model learning.
- A 2D-CNN-based regression model for defect interval localization. We reformulate the defect detection task from a conventional classification problem into a direct regression of defect interval boundaries. The model was designed to predict both the start and end positions of defects, enabling precise spatial localization.
- Integration of Tree Seed Algorithm (TSA) for hyperparameter optimization. To mitigate the risk of suboptimal solutions associated with manual tuning, we employ the TSA to jointly optimize key parameters, including both network architecture parameters and Mel spectrogram transformation settings.
- Comprehensive evaluation using multiple performance metrics. Model performance is assessed using the Mean Absolute Error (MAE), Root Mean Square Error (RMSE), Coefficient of Determination (R^2^), Intersection over Union (IoU), and hit rate, which enable a quantitative analysis of both prediction accuracy and localization consistency.

## 2. Construction of Localization Model for Cross-Sectional Structural Damage in Receiver Tubes

### 2.1. Time-Series Defect Identification Network Based on 1D-CNN

**Figure 2:**
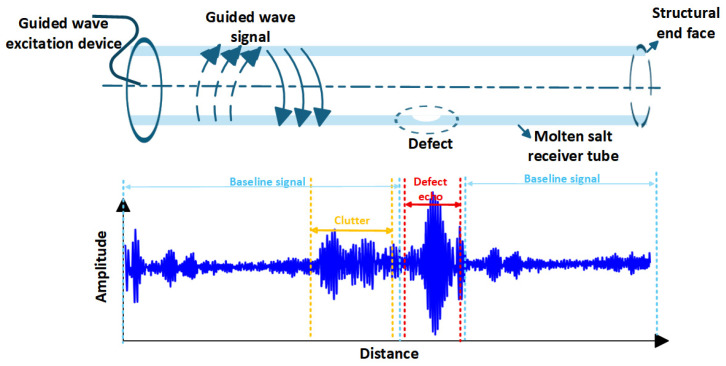
Schematic illustrating the reflection and dispersion characteristics of guided wave signals in defective molten salt receiver tubes.

**Figure 3:**
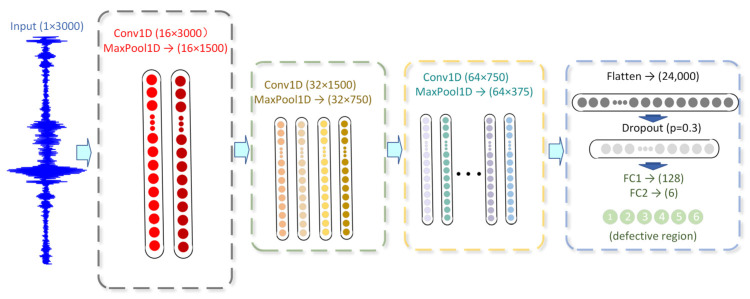
Architecture of a 1D-CNN network designed for time-series defect localization.

A standard 1D-CNN comprises an input layer, a sequence of convolutional and pooling layers, fully connected layers, and an output layer. To accommodate the reflection and dispersion characteristics inherent in guided wave signals arising from cross-sectional structural damage in molten salt receiver tubes (Figure 2), we propose the 1D-CNN architecture shown in Figure 3. This network employs a hierarchical convolutional architecture with progressively expanding receptive fields to extract multi-scale features. Additionally, Rectified Linear Unit (ReLU) activation functions and dropout mechanisms are integrated to enhance non-linear mapping capabilities and generalization performance [21,22].

**Figure 4:**
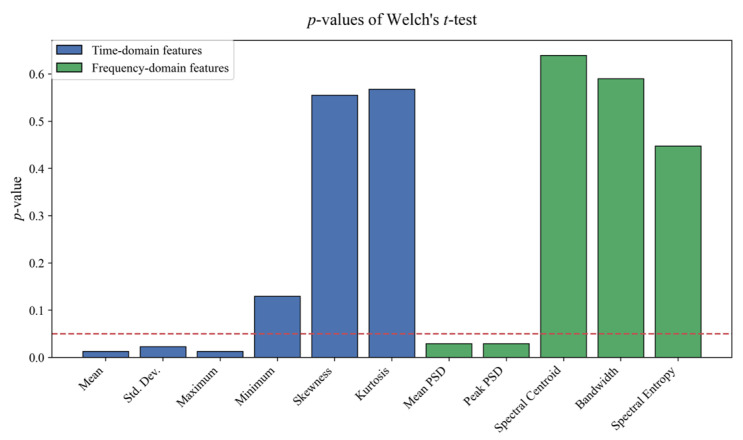
Comparative analysis of time–frequency features between stationary (baseline) and defect signals.

As shown in Figure 4, a differential feature analysis of the raw guided wave data reveals significant disparities between stationary and defect signals, not only in time-domain statistics but also in frequency-domain distributions. Statistical significance tests (*p*-values) on various time-domain metrics (e.g., standard deviation, extrema, mean) and frequency-domain metrics (e.g., mean peak power spectrum) confirm that features in both domains exhibit high discriminability. Hence, feature extraction based solely on time-domain information cannot capture the dispersion responses and spectral structures intrinsic to defects.

Consequently, while 1D-CNNs are appealing for time-series modeling, their ability to perceive global patterns and frequency-domain information is limited. This limitation is particularly pronounced in the long-duration guided wave signals characteristic of slender molten salt receiver tubes, where defect energy is often concentrated within specific frequency bands—information that a purely time-domain architecture cannot fully exploit. Spurred by this concern, this study introduces a time–frequency representation method to construct a 2D-CNN-based model. The developed approach aims to fuse multi-scale and multi-dimensional information to achieve high-precision localization of cross-sectional structural damage in slender molten salt receiver tubes.

### 2.2. Two-Dimensional Time–Frequency Transformation Strategy for One-Dimensional Guided Wave Signals

To augment the convolutional neural network’s capacity to capture the time–frequency characteristics of defects, we transform 1D guided wave signals into 2D image representations. Given the inherent dispersion characteristics and time–frequency distribution patterns of guided wave signals, the Mel spectrogram was selected as the primary method for two-dimensional representation. Furthermore, Gramian Angular Fields (GAF) [12] and the Wigner–Ville Distribution (WVD) [14] were incorporated into the experimental analysis as comparative baselines to substantiate the superiority of the Mel spectrogram.

#### Time–Frequency Representation via Mel Spectrogram

The Mel spectrogram generates a compact, robust 2D time–frequency through the application of a Short-Time Fourier Transform (STFT) to the time-domain signal, followed by a non-linear mapping of the frequency axis to the Mel scale [23]. This transformation aligns the spectral representation with human auditory perception, effectively emphasizing relevant frequency bands. STFT is mathematically formulated as [13]

(1) $$STFT\left\{x\right\}\left(t,f\right)=\sum_{n=-\infty }^{\infty }x[n]w[n-t]e^{-2\pi jfn}$$

where *w[n]* denotes the window function. The mapping relationship for the Mel scale is defined as

(2) $$\left\{\begin{array}{c} m=2595\log_{10}(1+\frac{f}{700})\\ f=700(10^{m/2595}-1) \end{array}\right.$$

where *m* represents the transformed Mel frequency, and f corresponds to the original frequency.

Mel spectrograms are employed as the primary time–frequency representation for guided wave signals. The core rationale lies in their non-linear frequency compression and multi-scale filter bank architecture. Given the dispersive nature of ultrasonic guided waves, where different frequency bands correspond to distinct propagation modes, defect-induced scattering or mode conversion is often localized around specific center frequencies. The Mel transform utilizes dense triangular filter banks in the low-frequency range to achieve high-resolution sampling, enabling the detection of subtle spectral shifts caused by defects. Simultaneously, logarithmic compression in the high-frequency range effectively suppresses random noise arising from heterogeneous salt films and coatings. Compared to STFT with its fixed resolution or Wavelet Transform with its reliance on empirical wavelet selection, the Mel spectrogram provides a more robust and compact adaptive frequency partitioning, facilitating the extraction of discriminative defect features by deep learning models in dispersive environments.

**Figure 5:**
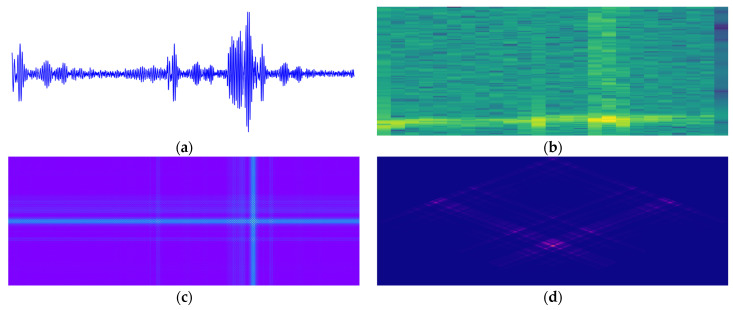
Results of transforming (**a**) a raw guided wave signal using three prominent methods: (**b**) the Mel spectrogram, (**c**) GAF, and (**d**) WVD.

Figure 5 illustrates the comparative visualization of three two-dimensional representation methods applied to guided wave signals from a defective receiver tube. Figure 5b reveals that the Mel spectrogram prioritizes low-frequency energy features, yielding a 2D image with a concise structure and concentrated information, promoting efficient feature extraction and model training. Conversely, the GAF (Figure 5c) emphasizes global morphological information but struggles to articulate local details and low-frequency energy distributions. Furthermore, WVD (Figure 5d) offers high-resolution time–frequency distributions that reveal local signal nuances but suffer from cross-term interference [12], which degrades the structural clarity of the image.

Given the trade-offs among information integrity, computational efficiency, and model compatibility, the Mel spectrogram balances performance and efficiency and was therefore adopted as the 2D representation of guided wave signals in this research.

### 2.3. Architecture of Mel+2D-CNN Defect Interval Regression Network

**Figure 6:**
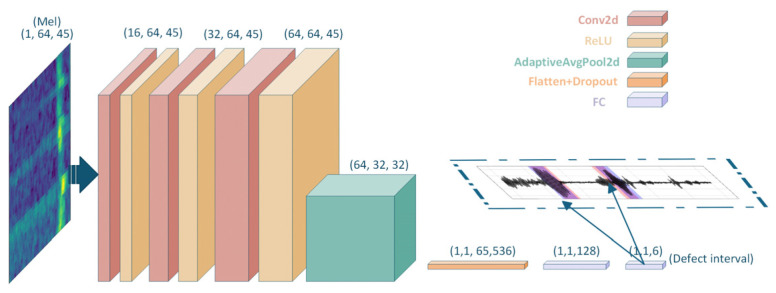
Schematic of the Mel+2D-CNN defect location regression network.

Leveraging the Mel spectrogram as the primary input representation, a defect interval regression network is established using a 2D-CNN architecture, as depicted in Figure 6. The network accepts single-channel grayscale images of size 1 × 64 × 45 as input. Structurally, it comprises a sequence of convolutional and pooling blocks that use 3 × 3 kernels to extract local time–frequency features. A ReLU activation function succeeds each convolutional operation to augment non-linear feature representation. After convolution and pooling, the resulting feature maps are flattened and propagated through fully connected layers to facilitate feature fusion and mapping. The output layer has a 6D linear activation function, corresponding to the start and end coordinates of up to three potential defects. This architecture allows the simultaneous regression of interval boundaries across multiple defects. During training, the Mean Squared Error (MSE) serves as the loss function, which, in conjunction with an appropriate optimization algorithm, drives the network to learn the complex mapping between two-dimensional Mel spectrogram patterns and defect intervals. The 2D-CNN model outputs six values, corresponding to the start and end sampling points of up to three defects. For samples containing fewer than three defects, the start and end positions of all non-existent defects are uniformly labeled as (0,0). During training, a Masked Mean Squared Error (Masked MSE) loss function is employed to automatically ignore the loss contribution from all (0,0) intervals, calculating errors only for actual defects. All defects are labeled in strict axial order from the proximal to the distal end of the transducer, ensuring the model learns a deterministic mapping relationship. During inference, all (0,0) invalid intervals in the output are filtered out, and the remaining results represent the predicted defect locations.

### 2.4. Hyperparameter Optimization via Tree Seed Algorithm (TSA)

The performance of deep neural networks depends on the optimal configuration of hyperparameters, including the learning rate, dropout probability, and neuron count in fully connected layers, as well as feature extraction parameters such as the frequency resolution and window length of the Mel spectrogram. The search space for these hyperparameters is typically high-dimensional and non-linear. Consequently, traditional methods such as grid search or manual tuning are often computationally inefficient and prone to local optima, thereby precluding the attainment of a global optimum. Hence, the TSA is employed to perform the global, adaptive joint optimization of the hyperparameters for the proposed Mel+2D-CNN regression network.

#### 2.4.1. Principles of TSA

The TSA process initiates by generating a population of trees within the defined search space:

(3) $${T_{i}=L}_{min}+rand(0,1)\times (H_{max}-L_{min})$$

where *Ti* denotes the initial position of the *i*-th tree, *L_min_* and *H_max_* represent the lower and upper bounds of the search space, respectively, and rand is a random variable. From the trees generated in Equation (3), the optimal position is identified based on the fitness function:

(4) $$B=ValLoss\left\{Mel\;CNN(T_{i})\right\}$$

where *B* signifies the optimal fitness value. The tree exhibiting the optimal fitness generates new seeds to propagate the search, utilizing one of the mechanisms described below:

(5) $$S_{i,j}=\left\{\begin{array}{c} T_{i}+\alpha_{i}*(T_{i}-T_{r}),\;rand<ST\\ T_{i}+\alpha_{i}*(B-T_{r}),\;otherwise \end{array}\right.$$

where *α_i_* is the step size factor, and *ST* is a control threshold that governs the transition between global exploration and local exploitation, thereby mitigating the risk of premature convergence to local optima.

#### 2.4.2. Optimization Objective

The TSA is applied to the joint optimization of seven critical hyperparameters governing the Mel+2D-CNN model. The objective function to minimize the validation loss is formulated as

(6) $$L_{val}=\frac{1}{N_{val}}\sum_{i=1}^{N_{val}}{\left\|{\hat{y}}_{i}-y_{i}\right\|}^{2}$$

This objective minimizes the Mean Squared Error (MSE) on the validation dataset. Table 1 reports the specific hyperparameters and their corresponding search intervals. In addition to the standard 2D-CNN network parameters, we also optimized specific Mel spectrogram hyperparameters. Notably, n_fft and hop_length jointly determine the time–frequency resolution of the damage signals from the molten salt receiver tubes. Parameter n_fft defines the analysis window size, which must be large enough to encompass the duration of multiple ultrasonic reflections and interferences within the tube wall. Meanwhile, hop_length dictates the step size and, consequently, the window overlap rate. An appropriate overlap is crucial to maintain temporal continuity and prevent the loss of damage-induced transient features—such as waveform distortions or abrupt phase shifts—between consecutive analysis frames. Finally, n_mels applies a non-linear frequency mapping that effectively compresses high-frequency components, thereby filtering out irrelevant environmental noise.

#### 2.4.3. Optimization Results and Analysis

**Figure 7:**
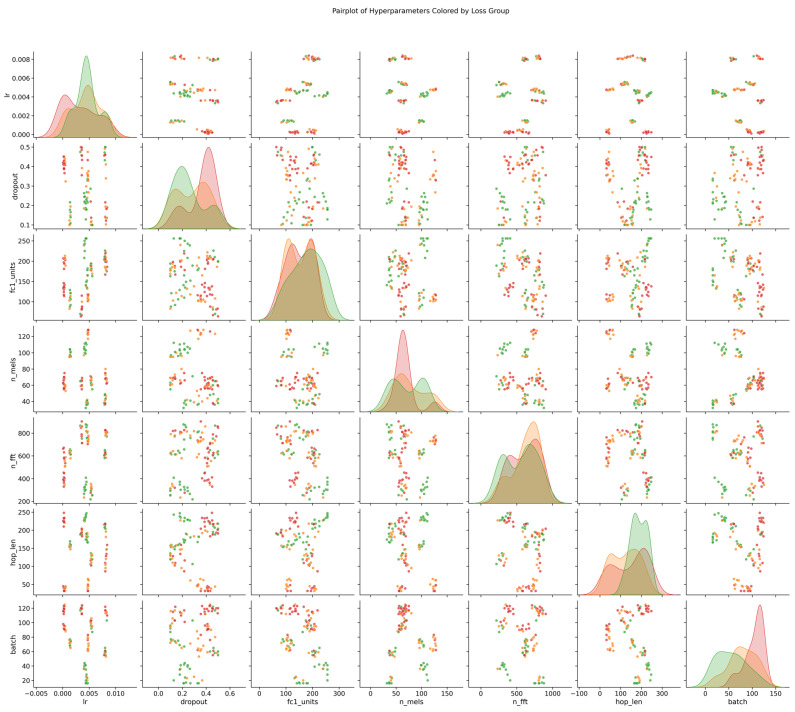
Scatter matrix visualization of the hyperparameter optimization process for the Mel+2D-CNN model.

The TSA executed multi-iteration optimization, yielding numerous hyperparameter combinations with varying performance metrics on the validation set. Figure 7 presents the optimization trajectory as a scatter matrix (pair plot) to visualize the sensitivity of model performance to these combinations.

Each data point in the above figure represents a specific hyperparameter configuration and its associated training outcome. The color gradient encodes the normalized loss value: red regions denote high loss (inferior performance), orange regions indicate moderate loss, and green regions signify low loss (superior performance). This visualization delineates the hyperparameter subspaces that correspond to optimal performance. The light-colored points (green) in the above figure correspond to the globally optimal hyperparameter set, listed in Table 2. Specifically, setting n_fft to 262 ensures adequate frequency resolution to capture the subtle characteristic frequency shifts caused by minor damage in the molten salt tube wall. Concurrently, a hop_length of 130 yields an overlap of approximately 50%, adhering to fundamental signal processing sampling principles. This configuration not only preserves temporal localization precision but also prevents the omission of transient defect signatures in the ultrasonic guided waves between successive analysis frames, thereby guaranteeing feature continuity. Furthermore, employing 62 Mel filter banks (n_mels) for non-linear mapping effectively facilitates the focused monitoring of damage-sensitive frequency bands. This configuration demonstrates an optimal trade-off between convergence velocity and generalization capability on the validation set.

## 3. Experimental Design and Data Analysis

### 3.1. Computational Environment and Configuration

The experimental setup involved a system running Windows 10 with 32 GB of RAM, with a 14th Gen Intel(R) Core(TM) i7-14700K, and an NVIDIA GeForce RTX 3080 with 10 GB of VRAM. The deep learning framework used was PyTorch 2.4.1, Python 3.9, and CUDA 12.1.

### 3.2. Experimental Dataset Acquisition and Augmentation

**Figure 8:**
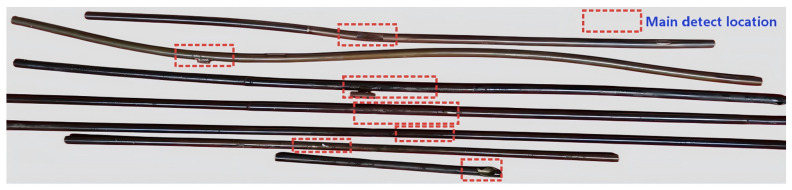
Photograph showing typical cross-sectional structural damage in molten salt receiver tubes.

The dataset utilized was derived from slender molten salt receiver tubes decommissioned from a tower-type molten salt Concentrated Solar Power (CSP) station (Figure 8). These tubes, with a diameter of 40 mm and lengths between 2 and 3 m, were made of Inconel 625 (density: 8440 kg/m^3^; elastic modulus: 210 GPa; Poisson’s ratio: 0.3).

**Figure 9:**
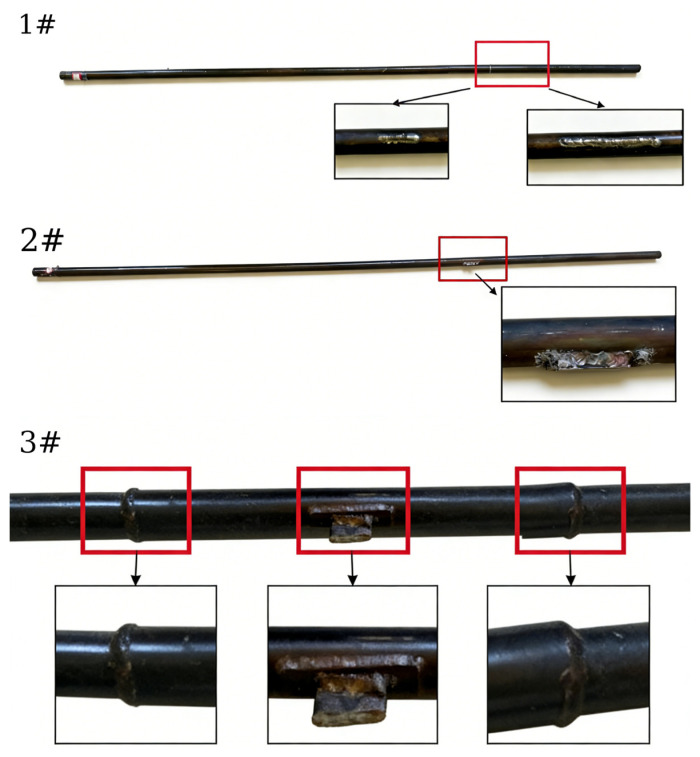
Photographs of representative defects in the receiver tubes: 1#longitudinal weld; 2#localized surface protrusion; 3#similar surface protrusion located between two adjacent butt welds.

Given that the first generation of Concentrated Solar Power (CSP) plants in China, specifically molten salt tower systems, have only been in operation for 5–10 years, the availability of naturally occurring defective receiver tubes is limited. Consequently, the specimens used in this study were sourced from decommissioned receiver tubes from the Delingha solar tower plant in Qinghai, China. These defects were artificially induced and encompass cracks, surface corrosion (minor), and welding defects. The welding defects primarily consist of longitudinal and butt weld flaws, with orientations predominantly aligned with the axial direction—a configuration highly sensitive to guided wave inspection. Figure 9 and Table 3 show the physical defect images of selected receiver tubes and their corresponding defect parameters, respectively.

**Figure 10:**
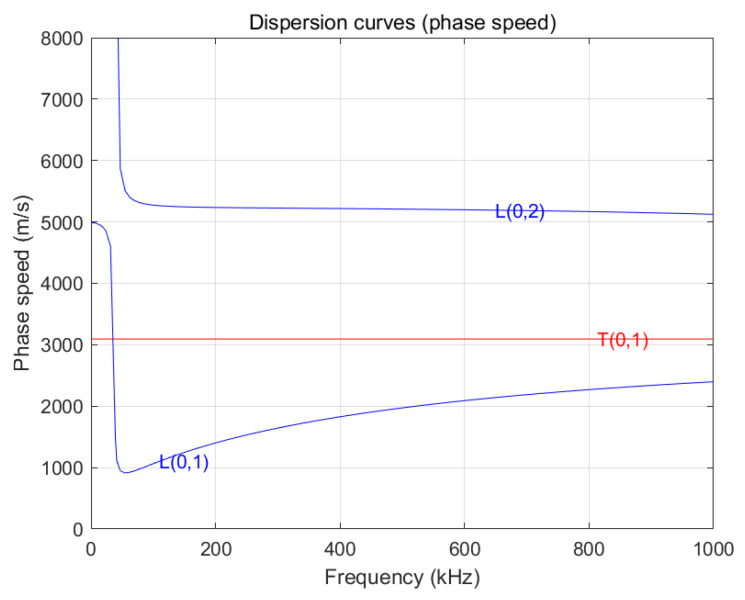
Group velocity dispersion curves for the ultrasonic guided waves in the receiver tubes.

Data were acquired using an ultrasonic guided wave testing system (Model: MSGW30). Guided by the group velocity dispersion curves depicted in Figure 10, the T(0,1) mode was selected for its near-zero dispersion characteristics, which ensure minimal waveform distortion and high signal stability. Theoretical analysis of the dispersion curves reveals that the T(0,1) mode is dominant within the 100–150 kHz range, where the excitation of higher-order modes is effectively suppressed, ensuring high modal purity. Based on this, an experimental excitation signal with a center frequency of 128 kHz and a bandwidth of ±20 kHz was utilized, as this configuration provides an optimal balance between the signal-to-noise ratio (SNR) and propagation stability.

In the context of localizing guided wave defect signals, the amplitude-correlation-offset (ACO) [24] decomposition method serves as a compelling alternative due to its exceptional physical interpretability and ability to perform basic localization with scarce data and no prior knowledge. Nevertheless, its heavy reliance on handcrafted features significantly limits its robustness when applied to complex signals, such as those encountered in molten salt receiver tubes. Such signals are typically plagued by severe modal aliasing—which can render the cross-correlation (C) ineffective—and high background noise that can easily obscure the amplitude (A). Under these conditions, ACO struggles to extract high-order non-linear dependencies. To overcome these limitations, we adopted a GAN-based data augmentation and deep learning approach, structuring our dataset as follows:

- Training and Validation Sets (Synthetic Data Only): The 3000 GAN-generated samples were partitioned into a training set (2400 samples) and a validation set (600 samples) at an 80/20 ratio, which were used exclusively for model parameter optimization and hyperparameter tuning.
- Test Set (Experimental Data Only): The 100 original experimental samples were strictly excluded from the training and tuning phases, serving solely as an independent hold-out test set to ensure an unbiased performance assessment. The detailed partitioning strategy is summarized in Table 4.

### 3.3. Model Performance Evaluation Metrics

To comprehensively assess the TSA+Mel+2D-CNN model’s capability to localize defect segments and delineate their boundaries, the experimental dataset was partitioned into training and test subsets adopting an 8:2 ratio. The model’s performance on the test set was quantified using a multi-dimensional evaluation framework, i.e., error metrics (Mean Absolute Error, Root Mean Square Error), goodness of fit (Coefficient of Determination), interval localization precision (Intersection over Union, Hit Rate), and a composite score. The specific metrics and their definitions are detailed below.

#### 3.3.1. Mean Absolute Error (MAE)

MAE quantifies the average magnitude of the difference between predicted and ground-truth values. It is defined as

(7) $$MAE=\frac{1}{n}\sum_{i=1}^{n}\left|y_{i}-{\hat{y}}_{i}\right|$$

where *y_i_* denotes the ground-truth value, *y_i_* represents the predicted value, and *n* is the total number of samples. A lower MAE indicates a higher proximity of predictions to the ground truth, signifying reduced error.

#### 3.3.2. Root Mean Square Error (RMSE)

RMSE calculates the square root of the average squared differences, thereby penalizing larger errors more heavily than smaller ones. This metric is particularly critical for evaluating the model’s sensitivity to outliers and is expressed as

(8) $$RMSE=\sqrt{\frac{1}{n}\sum_{i=1}^{n}(y_{i}-{\hat{y}}_{i}{)}^{2}}$$

#### 3.3.3. Coefficient of Determination (R^2^)

R^2^ assesses the goodness of fit, indicating the proportion of variance in the dependent variable that is predictable from the independent variable. It is formulated as

(9) $$R^{2}=1-\frac{\sum_{i=1}^{n}{\left(y_{i}-{\hat{y}}_{i}\right)}^{2}}{\sum_{i=1}^{n}{\left(y_{i}-{\bar{y}}_{i}\right)}^{2}}$$

where *y_i_* is the mean of the ground-truth values. An *R^2^* value approaching 1 indicates a strong model fit.

#### 3.3.4. Intersection over Union (IoU)

To validate the reliability of deep learning evaluation benchmarks, a transformation from the time-domain signal to the defect’s physical location is required. As illustrated in Figure 10, a rectangular crack (length ≈ 1.5 cm) was artificially introduced on a 1.0 m pipe specimen at 0.52 m from the proximal end. The Time-of-Flight (ToF) method (Equation (10)) was employed to map the time-domain signal to the physical defect location d:

(10) $$d=\frac{c_{p}\times \Delta t}{2}$$

where *C_p_* is the phase velocity of the guided wave (*C_p_* = 3000 m/s, derived from the dispersion curve in Figure 10), and Δ*t* represents the echo Time of Flight.

To evaluate the accuracy of the ToF-based spatial mapping, we compared the calculated defect positions with the actual measurements (Table 5). The results indicate that the localization error is consistently maintained within the range of 4–10%, demonstrating the engineering viability of the proposed labeling method. Furthermore, to enhance the reliability of the defect interval identification, manual verification of critical echoes was integrated into the signal processing workflow.

**Figure 11:**
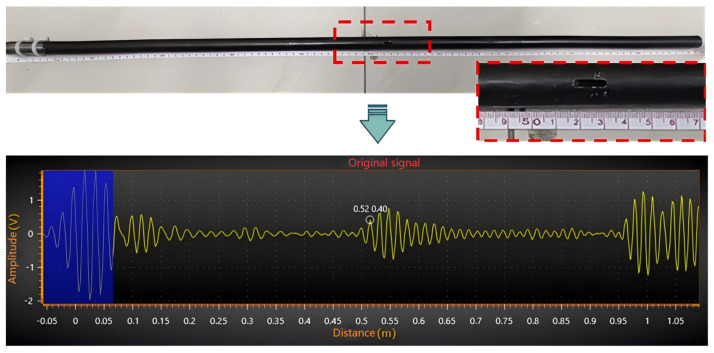
Waveform of the measured signal from a defective receiver tube.

The signal analysis in Figure 11 reveals a defect echo initiating at approximately 0.52 m, with an interval spanning 0.52–0.57 m. This corresponds closely with the actual physical location of the crack, confirming that the ToF-based localization error remains within acceptable engineering tolerances.

Consequently, defect intervals annotated via this method serve as the ground truth. Building on this, the Intersection over Union (IoU) is adopted as a core metric to precisely quantify the overlap between the predicted and ground truth intervals.

**Figure 12:**
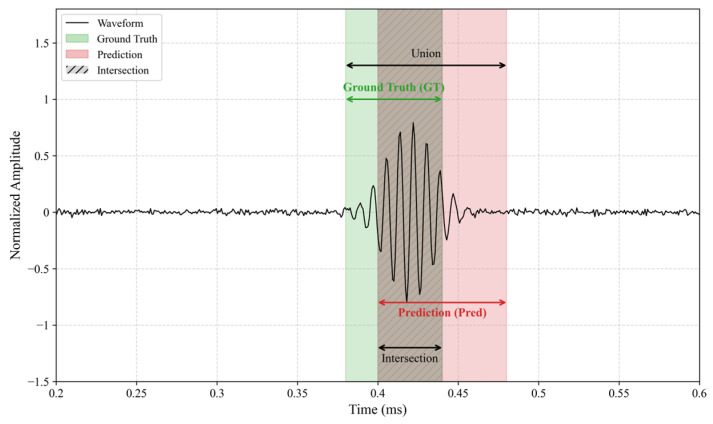
Schematic of Intersection over Union (IoU).

In defect interval identification, IoU measures the spatial overlap between the predicted and ground-truth segments, as depicted in Figure 12. *IoU* is formulated as

(11) $$IoU=\frac{Area(P\cap G)}{Area(P\cup G)}$$

where *P* and *G* denote the predicted and ground-truth intervals, respectively. The *IoU* ranges from [0, 1], with higher values indicating superior localization accuracy.

#### 3.3.5. Hit Rate (Accuracy)

To provide a multi-dimensional and comprehensive assessment of the localization accuracy for defects in molten salt receiver tubes, we adopted the Intersection over Union (IoU) as our primary evaluation metric. Drawing upon established conventions in object detection and the criteria for sample matching quality defined in seminal algorithms such as Fast R-CNN [25], we established three hierarchical IoU thresholds—0.3, 0.5, and 0.7—to characterize the model’s performance across varying levels of precision:

- Coarse Detection (Hit@0.3): A threshold of 0.3 is utilized to evaluate the model’s capability for initial defect coverage. This metric ensures that anomalous intervals are effectively captured during the preliminary screening phase, thereby minimizing the risk of false negatives.
- Standard Localization (Hit@0.5): Adhering to the mainstream benchmarks in object detection [26,27], an IoU > 0.5 is defined as a successful detection. This metric reflects the model’s robustness in maintaining reliable detection performance amidst complex industrial background noise.
- High-Precision Prediction (Hit@0.7): Drawing on criteria for positive sample assignment in high-precision localization and small-object detection studies, the Hit@0.7 metric is employed to rigorously evaluate the model’s accuracy in predicting defect boundaries (i.e., start and end positions).

#### 3.3.6. Comprehensive Weighted Score

For a holistic comparison across multiple metrics, a normalized weighted scoring method was introduced. The final comprehensive score (Score) is defined as

(12) $$Score=\sum_{i=1}^{M}\omega_{i}\cdot N_{i}$$

where *w_i_* is the weight assigned to the *i*-th metric, *N_i_* is the normalized value of that metric, and *M* is the total number of metrics evaluated.

## 4. Results and Discussion

### 4.1. Comprehensive Performance Evaluation

It is important to note that different performance metrics reflect distinct capabilities of the model. Mean Absolute Error (MAE) and Intersection over Union (IoU) hit rates serve as the primary metrics for the defect localization task, as they directly quantify the accuracy of the model in predicting defect positions and boundaries—factors of paramount importance in industrial non-destructive testing. Conversely, the Root Mean Square Error (RMSE) and Coefficient of Determination (R^2^) are considered secondary metrics; while they reflect the model’s ability to fit the overall signal distribution, their utility in guiding practical engineering localization is relatively limited.

**Figure 13:**
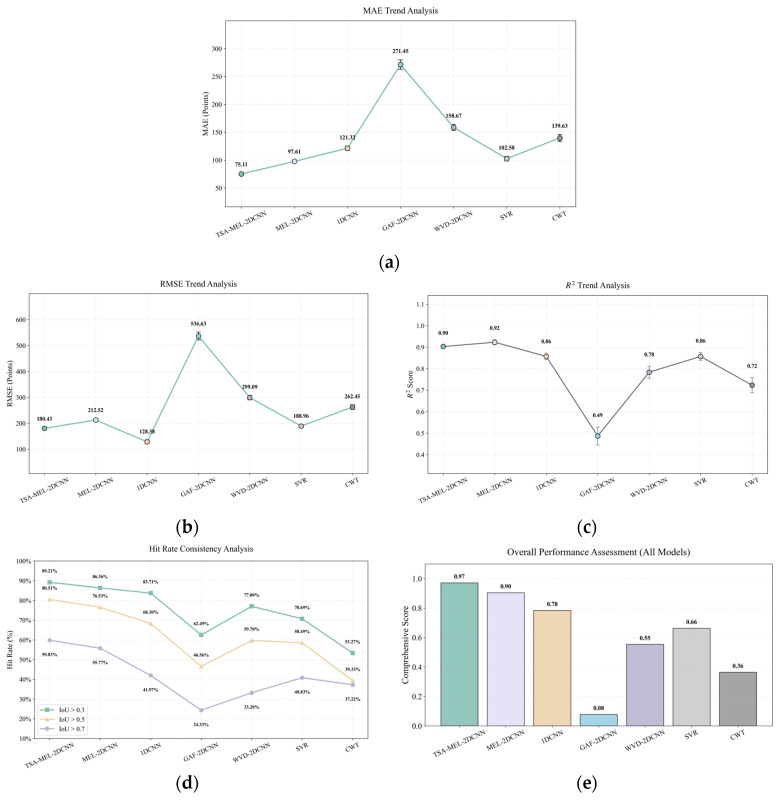
Comparative performance analysis of (**a**) MAE, (**b**) RMSE, (**c**) R^2^, (**d**) IoU threshold hit rate (0.3, 0.5, and 0.7), and (**e**) overall performance assessment across multiple metrics.

As shown in Figure 13, a dual-perspective evaluation was also conducted on the test set to rigorously assess the efficacy of the seven models (Mel+2D-CNN, 1D-CNN, GAF+2D-CNN, WVD+2D-CNN, and traditional methods (SVR and CWT)) on the defect segment localization and regression task. This included regression accuracy metrics (MAE, RMSE, R^2^) and hit-rate metrics (defect segment detection performance across varying IoU thresholds). Subsequently, a normalized weighting strategy was applied to derive a composite performance score.

Figure 13a illustrates the comparative results of the seven models in terms of the Mean Absolute Error (MAE), which directly quantifies the physical distance between predicted and ground-truth defect locations. The TSA-Mel-2DCNN model achieved the lowest MAE (75.11), representing an approximately 23% reduction compared to the unoptimized Mel-2DCNN (97.61). This improvement demonstrates that the Tree Seed Algorithm (TSA) mechanism effectively enhances the extraction of critical defect-related features. In contrast, traditional methods such as Support Vector Regression (SVR) and Continuous Wavelet Transform (CWT) yielded significantly higher errors of 102.58 and 139.63, respectively. From an engineering perspective, a lower MAE indicates a more precise localization of the defect center, which is crucial for minimizing unnecessary excavation or false-positive inspection areas.

Figure 13b presents the Root Mean Square Error (RMSE) results. As RMSE is highly sensitive to outliers, it serves as a key indicator of prediction stability. While 1D-CNN achieved the lowest RMSE (128.38), suggesting an advantage in suppressing large-scale error fluctuations through raw signal processing, the TSA-Mel-2DCNN (180.43) significantly outperformed other 2D time–frequency models, such as WVD (299.09) and GAF (536.63). This indicates that the integration of the TSA module not only improves average precision but also enhances the robustness of the regression process, effectively mitigating severe deviations from the ground truth.

Figure 13c displays the Coefficient of Determination (R^2^), which measures the model’s ability to interpret the distribution of defect locations. The Mel-2DCNN and TSA-Mel-2DCNN models achieved high goodness-of-fit values of 0.92 and 0.90, respectively, followed by 1D-CNN and SVR (both 0.86). Conversely, the GAF+2DCNN model yielded a score of only 0.48, suggesting that its feature mapping approach fails to capture the complex non-linear relationships between ultrasonic guided wave signals and defect positions. These results confirm that deep convolutional networks based on Mel spectrograms possess superior statistical fitting capabilities for non-stationary guided wave signals.

Figure 13d presents the hit rates at various IoU thresholds (0.3, 0.5, and 0.7). IoU serves as a rigorous metric for evaluating the overlap between predicted and actual defect intervals. The TSA-Mel-2DCNN consistently outperformed all other models, achieving a hit rate of 89.21% at IoU@0.3 and maintaining a robust rate of 59.83% even under the stringent IoU@0.7 condition. This significantly exceeds the performance of SVR (40.83%) and CWT (37.22%), demonstrating that time–frequency fusion features provide a distinct advantage in identifying precise defect boundaries, thereby meeting the requirements for high-reliability industrial NDT.

To provide a holistic performance assessment, Figure 13e presents a comprehensive score calculated via multi-metric normalized weighting. The TSA-Mel-2DCNN ranks first, driven by its dual advantages in localization accuracy (MAE) and boundary identification reliability (IoU). In contrast, while traditional baselines like SVR demonstrate moderate regression capabilities, they exhibit clear limitations in boundary localization under complex waveform distortion. Furthermore, the GAF and WVD methods underperformed across all metrics, likely due to the introduction of artifacts or information loss during the feature extraction process.

In conclusion, the performance disparities among the models highlight the complementary nature of their feature extraction mechanisms. Theoretically, the 1D-CNN excels in signal fitting for raw time-series data, as evidenced by its superior RMSE and R^2^ scores. However, the TSA+Mel+2D-CNN model, leveraging Mel time–frequency mapping and intelligent optimization, achieves more precise defect localization, leading to improvements in MAE and IoU. This confirms that integrating multi-scale time–frequency representations with an adaptive hyperparameter search strategy effectively compensates for the limitations of traditional 2D models regarding regression stability, thereby achieving optimal performance in core localization metrics for comprehensive defect diagnostic tasks.

### 4.2. Ablation Studies

To further clarify the individual contribution of each core module, we conducted a systematic ablation study under a unified experimental framework, as summarized in Table 6. This analysis evaluates the impact of feature representation, network architecture, and hyperparameter optimization strategies.

We compared three 1D-to-2D signal transformation methods—Mel spectrogram, GAF, and WVD—within the same 2D-CNN framework. As shown in Table 6, the Mel spectrogram-based model consistently outperforms the others across all metrics, particularly in MAE and IoU. This suggests that the Mel spectrogram more effectively captures the time–frequency energy distribution of guided wave signals, thereby enhancing localization accuracy. In contrast, the GAF and WVD methods appear to introduce significant information loss or structural distortion, leading to increased regression errors and degraded localization performance.

A comparison between the 1D-CNN and Mel+2D-CNN models reveals that while 1D-CNN performs competitively in terms of RMSE, it is significantly outperformed by the Mel+2D-CNN in localization precision (IoU). This indicates that relying solely on 1D time-domain features is insufficient for precise defect interval identification. Conversely, the 2D-CNN architecture, when combined with Mel spectrogram representations, better captures local time–frequency structural information, resulting in superior defect boundary detection.

The impact of the TSA optimization strategy is evident when comparing Mel+2D-CNN with TSA+Mel+2D-CNN. The introduction of TSA leads to comprehensive performance gains: the MAE decreases significantly from 97.61 to 75.11, while the IoU@0.3, IoU@0.5, and IoU@0.7 metrics improve from 86.36% to 89.21%, 76.53% to 80.51%, and 55.77% to 59.83%, respectively. These improvements demonstrate the efficacy of the TSA mechanism in enhancing predictive accuracy. This success is attributed to the TSA’s ability to perform a global search within the high-dimensional parameter space, enabling synergistic optimization of both Mel transform parameters and 2D-CNN structural hyperparameters.

In summary, the ablation study confirms that the model’s superior performance is not merely the result of additive module stacking, but rather the synergistic integration of Mel spectrogram feature representation, 2D convolutional modeling, and TSA-based global optimization. These findings empirically validate the rationality and effectiveness of the proposed framework.

### 4.3. Robustness, Reproducibility, and Computational Efficiency Analysis

**Figure 14:**
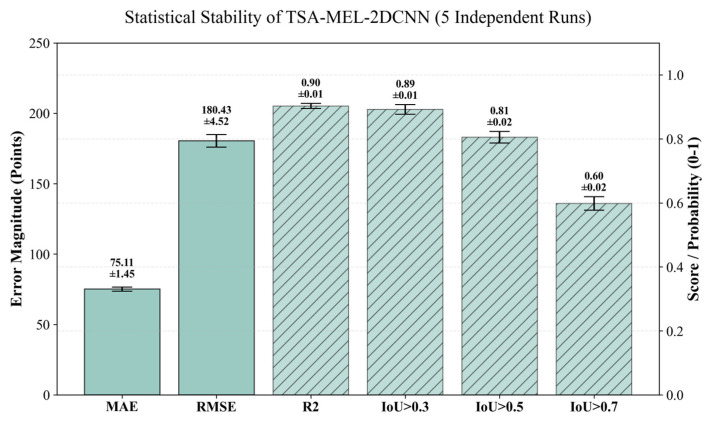
Bar chart showing the performance metrics of the TSA-MEL-2DCNN model with error bars representing the standard deviation across five independent runs.

To ensure the reproducibility and statistical significance of our findings, all models were subjected to five independent training and testing cycles, each initialized with a distinct random seed (e.g., 42, 123, 456, 789, and 1011). Rather than relying on single-run results, we report the mean and standard deviation across these five iterations, as illustrated in Figure 14. The inclusion of error bars demonstrates the consistent performance of the TSA-MEL-2DCNN model across all evaluation metrics. This consistency confirms that the observed performance gains are statistically robust and not attributable to stochastic variations in weight initialization.

Furthermore, to evaluate the deployment feasibility of the proposed framework for real-time structural health monitoring (SHM), we conducted a quantitative analysis of inference latency using an NVIDIA RTX 3080 GPU. The experimental results demonstrate that the TSA-MEL-2DCNN model achieves an average inference time of only 0.26 ms per ultrasonic guided wave signal (Table 7). This sub-millisecond processing speed, combined with the model’s reference-free nature, underscores its capability to meet the stringent computational demands of near real-time industrial inspection.

### 4.4. Impact of Label Uncertainty on Performance Metrics

The ground-truth labeling for defects in this study is conducted in two stages: first, the time intervals corresponding to defect echoes (i.e., the start and end sampling points) are manually identified within the signal; subsequently, these temporal positions are converted into physical distances along the pipeline using the Time-of-Flight (ToF) formula. Empirical validation reveals a 4–10% relative error between the physical positions derived from ToF calculations and the actual physical locations of the defects. This discrepancy is an inherent systematic error associated with the ToF method in ultrasonic guided wave testing.

It is important to clarify that the performance metrics reported in this paper, such as MAE and IoU, are calculated directly based on the sampling point positions within the signal. As these metrics are derived prior to the ToF conversion, they remain unaffected by the aforementioned physical distance errors. Consequently, the model’s predictive accuracy at the signal level is robust and reliable, with an MAE of 75.11 sampling points accurately reflecting the model’s capability to localize defect echoes.

The 4–10% error inherent to the ToF method is only superimposed when the model’s predictions are converted into physical locations for practical engineering applications. Furthermore, since a consistent ToF conversion standard is applied across all evaluated models, the performance comparisons between them remain fair and valid.

## 5. Conclusions

This study addresses the critical challenge of intelligently localizing cross-sectional structural damage in molten salt receiver tubes of tower-type CSP stations by proposing a novel hybrid framework, TSA+Mel+2D-CNN. Our approach combines multi-scale time–frequency feature extraction with swarm intelligence optimization. Notably, to mitigate the non-stationarity inherent in guided wave signals under complex operational conditions, a two-stage neural network architecture was developed: a 1D-CNN for rapid coarse detection, followed by a Mel spectrogram-based 2D-CNN for fine-grained boundary regression. The integration of the TSA for adaptive hyperparameter tuning further ensures the system’s precision and stability.

A comparative analysis of an augmented dataset highlights distinct performance characteristics among the evaluated models. While the 1D-CNN demonstrates marginal superiority in signal-fitting metrics (RMSE, R^2^) due to its direct temporal modeling, the TSA+Mel+2D-CNN model exhibits a decisive advantage in localization precision (IoU). In contrast, GAF and WVD-based methods underperformed, likely attributable to the degradation of fine temporal details during image transformation. Significantly, the proposed TSA+Mel+2D-CNN model achieves an optimal equilibrium across all performance dimensions. It minimizes the MAE to 75.11 and maintains a robust fit (R^2^ = 0.90). Most critically, it demonstrates superior robustness in defect interval localization, attaining hit rates of 89.21% (IoU > 0.3) and 80.51% (IoU > 0.5), thereby validating its efficacy in pinpointing minute defect boundaries.

In conclusion, the fusion of Mel spectrograms with 2D-CNN architectures offers a robust compromise between high-dimensional feature representation and computational efficiency. The adaptive optimization provided by TSA further refines the model’s sensitivity to nuances in the data distribution. This methodology significantly improves the precision of ultrasonic-guided-wave-based defect localization, effectively circumventing the limitations of conventional techniques in high-noise environments. Consequently, it presents a highly viable, engineering-grade solution for the structural health monitoring and assessment of receiver tubes operating under extreme thermal stress.

Although the proposed TSA-Mel-2DCNN model achieves superior performance in defect localization accuracy and overall robustness, several limitations remain that should be addressed. First, the model was trained specifically on ultrasonic guided wave signals from receiver tubes in solar thermal power plants, focusing on specific materials (e.g., Inconel 625), structural dimensions (e.g., pipe diameters of 19–60 mm), and typical defect types (e.g., weld defects, cracks, and corrosion). Consequently, the model’s performance in extreme operating conditions or across different structural and material configurations requires further validation. Given the current constraints on experimental sample size and defect diversity, the model necessitates further refinement before widespread industrial deployment.

In future work, we intend to enhance the model’s cross-scenario adaptability by incorporating small-scale target-domain data for fine-tuning or by employing few-shot transfer learning techniques. Furthermore, we plan to integrate material and structural parameters as auxiliary inputs to improve the model’s generalization capability across diverse industrial environments.

## Figures and Tables

**Table 1:** Hyperparameters and search ranges for the Mel+2D-CNN model.

Hyperparameter Name	Variable	Value Range
Learning rate	lr	[0.0001, 0.01]
Dropout probability	dropout	[0.1, 0.5]
FC layer neurons	fc1_units	[64, 256]
Mel filter count	n_mels	[32, 128]
FFT window size	n_fft	[128, 1024]
Hop length	hop_length	[32, 256]
Batch size	batch_size	[16, 128]

**Table 2:** Optimal hyperparameter configuration for the Mel+2D-CNN model.

Hyperparameters	Optimal Value
lr	0.000948
dropout	0.1
fc1_units	241
n_mels	62
n_fft	262
hop_length	130
batch_size	38

**Table 3:** Defect parameters of selected defective receiver tubes.

No.	Representative Defect	Axial Length (mm)	Circumferential Length (mm)
1#	Corrosion 1-1	30.54	21.44
	Corrosion 1-2	76.12	20.68
2#	Longitudinal weld defects 2-1	39.94	32.24
3#	Butt weld defects 3-1	38.40	-
	Cracks 3-2	73.46	35.76
	Butt weld defects 3-3	26.38	-

**Table 4:** Dataset partitioning strategy.

Dataset Type	Source	Quantity	Purpose
Training Set	Synthetic Data	2400	Model parameter updates, feature learning
Validation Set	Synthetic Data	600	Hyperparameter tuning, overfitting prevention
Test Set	Experimental Data	100	Final accuracy assessment, real-world performance validation

**Table 5:** Defect detection locations and corresponding errors.

No.	Representative Defect	Detected Distance (mm)	Actual Distance (mm)	Relative Error
1#	Corrosion 1-1	430.0	390.3	10.18%
	Corrosion 1-2	520.0	501.0	4.21%
2#	Longitudinal weld defects 2-1	1280.0	1361.0	6.00%
3#	Butt weld defects 3-1	350.0	380.5	8.01%
	Cracks 3-2	1001.0	1066.0	6.19%
	Butt weld defects 3-3	1543.0	1609.1	4.11%

**Table 6:** Ablation study on different model components and feature representations. The best performance for each model configuration is highlighted in bold.

Model	MAE ↓	RMSE ↓	R^2^ ↑	IoU@0.3 ↑	IoU@0.5 ↑	IoU@0.7 ↑
TSA+MEL+2D-CNN	**75.11**	180.43	0.90	**89.21%**	**80.51%**	**59.83%**
MEL+2D-CNN	97.61	212.52	**0.92**	86.36%	76.53%	55.77%
1D-CNN	121.32	**128.38**	0.86	83.71%	68.30%	41.97%
GAF+2D-CNN	271.45	536.63	0.49	62.49%	46.56%	24.33%
WVD+2D-CNN	158.67	299.09	0.78	77.00%	59.70%	33.20%

**Table 7:** Comparison of statistical performance and computational efficiency across different models (mean ± standard deviation).

Models	MAE	R^2^	Hit Rate (IoU@0.7)	Average Inference Time (ms)
TSA-MEL-2DCNN	75.11 ± 1.5	0.90 ± 0.01	59.83% ± 2.1%	0.26
MEL-2DCNN	97.61 ± 2.4	0.92 ± 0.01	55.77% ± 1.8%	0.24
1DCNN	121.32 ± 3.8	0.86 ± 0.02	41.97% ± 3.5%	0.35
SVR	102.58 ± 4.2	0.86 ± 0.02	40.83% ± 2.9%	1.40
CWT	139.63 ± 6.5	0.72 ± 0.04	37.22% ± 4.1%	0.39
GAF-2DCNN	271.45 ± 8.1	0.49 ± 0.05	24.33% ± 5.2%	0.15
WVD-2DCNN	158.67 ± 5.4	0.78 ± 0.03	33.20% ± 4.7%	0.16

## Data Availability

The data presented in this study are available upon request from the corresponding author. The data are not publicly available due to privacy restrictions.

## Abbreviations

The following abbreviations are used in this manuscript:

NDT	Non-destructive testing
CSP	Concentrated Solar Power
1D-CNN	One-dimensional convolutional neural network
TSA	Tree Seed Algorithm
MAE	Mean Absolute Error
IoU	Intersection over Union
